# Fall incidence in Germany: results of two population-based studies, and comparison of retrospective and prospective falls data collection methods

**DOI:** 10.1186/1471-2318-14-105

**Published:** 2014-09-20

**Authors:** Kilian Rapp, Ellen Freiberger, Chris Todd, Jochen Klenk, Clemens Becker, Michael Denkinger, Christa Scheidt-Nave, Judith Fuchs

**Affiliations:** Department of Clinical Gerontology, Robert-Bosch-Hospital, Stuttgart, Germany; Institute of Epidemiology and Medical Biometry, Ulm University, Ulm, Germany; Institute for Biomedicine of Aging, Friedrich-Alexander University Erlangen-Nürnberg, Erlangen, Germany; School of Nursing, Midwifery and Social Work, University of Manchester, and Manchester Academic Health Sciences Centre, Manchester, United Kingdom; Agaplesion Bethesda Clinic, Ulm, Germany; Department of Epidemiology and Health Monitoring, Robert Koch Institute, Berlin, Germany

**Keywords:** Accidental falls, Independent living, Epidemiology, Population-based

## Abstract

**Background:**

Fall incidence differs considerably between studies and countries. Reasons may be differences between study samples or different assessment methods. The aim was to derive estimates of fall incidence from two population-based studies among older community-living people in Germany and compare retrospective and prospective falls data collection methods.

**Methods:**

Data were derived from the 2008–11 wave of the German health interview and examination survey for adults (DEGS1), and the Activity and Function of the Elderly in Ulm study (ActiFE-Ulm). Data collection took place in community facilities (DEGS1) or participants’ homes (ActiFE-Ulm). Participation rates were 42% (newly recruited) and 64% (panel component) in DEGS1 and 19.8% in ActiFE-Ulm. Self-report retrospective fall data covering the previous 12 month period in DEGS1 and ActiFE-Ulm were collected, but only ActiFE-Ulm used prospective 12 month fall calendars. The incidence of ‘any fall’ and ‘recurrent falls’ were calculated for both methods.

**Results:**

Fall rates increased with age in men but not women. The ActiFE-Ulm prospectively assessed incidence (95% confidence interval) in women and men aged 65- < 90 years were 38.7 (36.9-40.5) and 29.7 (28.1-31.3) fallers/year and 13.7 (12.5-14.9) and 10.9 (9.9-12.0) recurrent fallers/year, respectively. Retrospective and prospective fall incidence in ActiFE-Ulm did not differ.The retrospectively assessed incidence of ‘any fall’ among persons 65- < 80 years were significantly lower in DEGS1 than ActiFE-Ulm (women: 25.7% (22.4-29.2) versus 37.4% (34.8-39.9); men: 16.3% (13.6-19.3) versus 28.9% (26.6-31.1). Retrospective incidence estimates of recurrent falls were similar in both studies for women (10.4% (8.3-12.9) versus 10.2% (8.5-11.8)) and men (6.1% (4.3-8.5) versus 8.4% (7.1-9.8)).

**Conclusion:**

Both studies were population-based, but retrospective self-reported fall incidence differed between studies. Study design influences retrospective reported fall incidence considerably. Costly collection of prospective data gives similar rates to the cheaper retrospective report method.

## Background

Assessing and reporting population-based incidence of falls is on the face of it straightforward. A number of epidemiologic studies [1–6] and a number of fall prevention trials [7] have reported fall incidence for different populations and have been reviewed elsewhere [8, 9]. Several of these studies (and their fall incidence estimates) are particularly often cited in comments, introductions or overviews [10–12]. Fall incidence, however, differs considerably between studies. In community-living persons aged 65 years and above, for example, fall incidence lower than 20% and higher than 30% has been reported [1, 13, 14]. One potential reason for these differences is that the study samples from which the incidence rates were derived represent different populations with different fall risks. They may be convenience samples, trial populations, subpopulations such as residents from nursing homes or participants of epidemiological cohorts.

In order to obtain accurate fall incidence rates, representative population-based samples with high participation rates are required. In western industrialised countries, however, high participation rates are increasingly difficult to achieve [15]. Despite considerable effort, participation rates in many studies remain unsatisfactory. This results in selection bias in participants, which is particularly problematic in the field of falls epidemiology since typical selection criteria for older people may be strongly associated with fall risk. Persons with mobility restrictions, for example, may decline participation if they have to go to a study centre. Cognitively impaired people may be generally underrepresented in studies since they are not able to respond to a study invitation or to complete a falls calendar regularly.

Furthermore, estimates of fall incidence in the literature are based on different assessment methods. A consensus expert meeting recommended prospective daily recording and adequate surveillance of documentation and ascertainment of details of falls at least once a month over lengthy periods (normally 6–12 months) [16]. But this is time-consuming and costly and often only feasible in relatively small and possibly unrepresentative samples. Therefore, surveys and epidemiologic studies frequently apply retrospective assessments, which may be biased by poor recollection of past falls. In publications fall rates are reported for different time periods (4 weeks, 3 months, 12 months), are calculated by different methods (incidence of fallers, incidence of frequent fallers, incidence rates), or are presented stratified by different age-categories (5- or 10-year strata) which further complicates comparison. For Germany, population-based data about fall incidence are rare. Only one study reported fall incidence so far, based on a retrospective telephone survey [17].

Our study presents fall incidence in community-living German women and men aged 65 years and older, stratified by five year age-group. Data were derived from (i) a national survey and (ii) a geriatric cohort study performed in southern Germany. Both studies aimed to be population-based. Additional objectives of this publication are to describe the influence of a) different study approaches and b) retrospective and prospective assessment methods on the reported incidence of falls.

## Methods

The data for our analyses were derived from (i) the German health interview and examination survey for adults (DEGS1) and (ii) the Activity and Function of the Elderly in Ulm study (ActiFE-Ulm). Both studies were undertaken in Germany and are population-based.

DEGS1 is part of the continuous health monitoring system in Germany and was conducted by the Robert Koch Institute between November 2008 and December 2011 [18]. It was designed to provide nationally representative health data for the population 18–80 years of age. For Germany this was the first time that falls were assessed as part of a national health examination survey in the 65–80 year age groups.

The ActiFE-Ulm study is a cohort study of older people (65- <90 years), located in Ulm and adjacent regions in Southern Germany. The baseline recruitment took place between March 2009 and April 2010 [19].

The study protocols of DEGS1 and ActiFE-Ulm were approved by the Charité-Universitätsmedizin Berlin ethics committee in September 2008 (No. EA2/047/08) and the Ulm University ethics committees in January 2009 (No. 318/08), respectively. Both studies are more fully described elsewhere [18–20]. Here we focus on those aspects of the study designs of direct relevance for this paper.

### Recruitment of the study population and participation rates

DEGS1 has a two-stage stratified cluster sampling design [20]. Primary sampling units were the communities. Within these units, random samples of individuals, stratified by 10-year age group, were drawn from local population registers. Persons who had already participated in the 1998 national health interview and examination survey (GNHIES98) [21] were invited to take part in DEGS1, provided they had agreed to be recontacted. In order to maintain representativeness at the population level, additional individuals from communities involved in the previous survey (GNHIES98) and from additional communities were sampled so as to replace the number of participants expected to decline participation in DEGS1 or to be lost to follow-up. Eligible persons were invited to participate in the survey by letter. An information booklet described the study objectives, procedures and logistics. In order to optimise response rates participants were offered a small monetary incentive, and survey activities were publicised in the media. Persons who agreed to participate were contacted by telephone. Individual follow up was undertaken with persons who had not responded to the invitation letter within four weeks. Exclusion criteria were insufficient language proficiency or inability to provide informed consent. Persons willing to participate but facing organisational problems (e.g. lack of transportation, time constraints) were offered transportation and flexible scheduling, such as appointments early in the morning or in the evening. Data assessment took place in community-owned facilities and was performed by peripatetic study teams.

Present response rates were higher among GNHIES98 participants (64%) compared to newly sampled individuals (42%).The response rate in the GNHIES98 in 1998 had been 61%. Fall data from 1013 women and 973 men aged 65- <80 years were available for analysis.

In the ActiFE-Ulm study postal addresses of community-living people were obtained from the local authority registry office. The sample was stratified for sex and age in order to achieve comparable numbers in the different age- sex-groups. Up to three invitation letters were sent out to each person sampled. If the person agreed to participate an examination and interview were performed by a physician and a study nurse within 3 visits at the participant’s home. Residents of nursing homes, and persons with severe deficits in cognition, vision, hearing and persons with insufficient German language were excluded. Some 1,506 eligible individuals agreed to participate and underwent baseline assessments (participation rate 19.8%). Data from 600 women and 788 men with complete information about prospectively and retrospectively assessed falls were included in the analysis.

### Definition and assessment of falls

Falls and their measurement were broadly defined in both the DEGS and ActiFE-Ulm studies following the ProFaNE definition and procedures [16, 22], and are reported in this paper in terms of a single fall, or recurrent falls (two or more falls) during the 12 month recording or exposure period (either retrospective or prospectively collected).

In DEGS1 the history of falls within the past 12 months (one fall, two falls or more) was assessed by a standardised self-completed questionnaire in participants 65 years and older (retrospective assessment) [18]. The question explicitly defined falls by reference to fall, stumble or slip and coming to rest on the floor or lower surface.

In the ActiFE-Ulm study falls were assessed prospectively over 12 months using weekly fall calendars. Instructions for participants defined falls by referring to coming to rest on the floor or lower surface in for both prospective and retrospective assessments of the ActiFE-Ulm study. Every three months the completed calendar records were sent back to the study centre. Participants were telephoned if calendars were not returned or if information was incomplete about falls (prospective assessment). In addition participants were asked by the study nurses at the baseline assessment about their fall history in the past 12 months (any fall; number of falls) (retrospective assessment). Therefore, reported retrospective and prospective falls did not refer to the same time periods.

### Statistical analysis

Fall data were summarised as recommended by ProFaNE [22] using number of non-fallers/single fallers/multiple fallers per person year.

For the prospective ActiFE-Ulm data, the incidence of ‘any fall’ was calculated by the number of fallers in the exposure period divided by the number of person-years exposure, since not all participants had an exposure time of one year. ‘Recurrent falls’ was defined as two or more falls within the exposure period and the incidence was calculated as per any fall. Fall incidence rates are presented stratified by age groups (5-year categories) and sex.

For the retrospective ActiFE-Ulm and DEGS1 data the incidence calculated was based simply on the number of falls reported for the previous 12 months divided by the number of respondents, likewise, for recurrent falls this was the number of persons reporting two or more falls. DEGS1 did not collect data for the two highest age groups (80- < 85 years; 85- < 90 years). In order to present representative data for the combined age-groups (65- < 80 years; 65- < 90 years) the age-specific fall incidence rates were weighted according to the age distribution of the German standard population (2010) [23]. Weighting factors for DEGS1 considered sampling, response and continued participation probabilities as described elsewhere in detail [18]. The 95% confidence intervals of rates are presented but no confirmatory inferential statistical testing was performed.

## Results

### Fall incidence

In both women and men aged 65–80 years the retrospectively assessed incidence of ‘any fall’ was significantly lower in the DEGS1 than in the ActiFE-Ulm study (25.7% (95% CI: 22.4-29.2%) vs. 37.4% (95% CI: 34.8-39.9%) fallers/year in women and 16.3% (95% CI: 13.6-19.3%) vs. 28.9 (95% CI: 26.6-31.1%) fallers/year in men) (Table 1). In the ActiFE-Ulm study the estimates of the retrospectively and prospectively assessed incidence in the two lower age-groups were similar whilst in the higher age groups (≥75 years) a tendency towards higher incidence in the retrospective assessment was observed.

**Table 1:** **Incidence rates of ‘any fall’ in participants of the German health interview and examination survey for adults (DEGS1) and the Activity and Function of the Elderly in Ulm study (ActiFE-Ulm)**

	Database: DEGS1		Database: ActiFE Ulm		
		*Retrospective assessment (12 months)*			*Prospective assessment (12 months)*
Age (years)	Number of participants	Incidence rate of any fall ^†^	Number of participants	Incidence rate of any fall ^†^	Incidence rate of any fall ^†^
**Women**					
65- < 70	403	24.4 (19.8-29.7)	162	31.5 (24.4-39.2)	39.9 (32.2-47.9)
70- < 75	416	25.6 (20.4-31.7)	183	36.1 (29.1-43.5)	36.5 (29.5-44.0)
75- < 80	200	27.5 (21.0-35.1)	110	46.4 (36.8-56.1)	38.5 (29.3-48.5)
80- < 85	-	-	86	40.7 (30.2-51.8)	37.5 (26.9-49.0)
85- < 90	-	-	59	44.1 (31.2-57.6)	42.2 (28.3-57.0)
Total*					
65- < 80	1019	25.7 (22.4-29.2)	455	37.4 (34.8-39.9)	38.2 (35.6-40.8)
65- < 90	-	-	600	38.9 (37.1-40.6)	38.7 (36.9-40.5)
**Men**					
65- < 70	396	13.1 (9.2-18.3)	170	22.9 (16.9-30.0)	25.9 (19.4-33.3)
70- < 75	402	16.6 (12.4-22.0)	240	30.0 (24.3-36.2)	31.2 (25.2-37.6)
75- < 80	181	21.1 (14.2-30.1)	111	36.0 (27.1-45.7)	27.4 (19.2-36.9)
80- < 85	-	-	203	36.9 (30.3-44.0)	33.5 (26.9-40.6)
85- < 90	-	-	64	46.9 (34.3-59.8)	38.0 (25.5-51.7)
Total*					
65- < 80	979	16.3 (13.6-19.3)	521	28.9 (26.6-31.1)	28.3 (26.1-30.6)
65- < 90	-	-	788	31.2 (29.6-32.7)	29.7 (28.1-31.3)

*Incidences weighted according to the age distribution of the German standard population (2010).

^†^fallers/100-person-years exposure; 95% confidence interval.

The prospectively assessed incidence rates in women and men aged 65- < 90 years were 38.7% and 29.7% fallers/year, respectively. Somewhat more than 40% of those with a prospectively assessed fall reported also at least one fall in the previous year (data not shown).

In both datasets (DEGS1 and Active-Ulm) increasing falls were observed with increasing age in men. In women, however, fall rates were not so clearly associated with age, as the women show a peak in the 75–80 years age range. Except for the highest age-categories fall incidence was higher in women than in men although confidence intervals overlap in every age strata (Table 1).

The incidence of recurrent falls in women was similar in both the DEGS1 and Active-Ulm datasets regardless of retrospective or prospective assessment with confidence intervals overlapping (Table 2). In men incidence of recurrent falls was again broadly similar with confidence intervals again overlapping. In men incidence of recurrent falls increased particularly in the highest age-group, whilst in women no clear association of fall rates with age was observed, although the confidence intervals for these oldest age groups are wide. Since not all participants completed 12 month follow up we conducted a sensitivity analysis to test if data were reliable for recurrent falls when based on less than 12 months follow up. This sensitivity analysis gave identical results, except in the oldest group of men (3.5% lower but well within confidence intervals), suggesting that drop out and recurrent falls may be related in this group. About 30% of the recurrent fallers (prospective assessment) experienced more than one fall in the previous year (data not shown).

**Table 2:** **Incidence rates of ‘recurrent falls’ in participants of the German health interview and examination survey for adults (DEGS1) and the Activity and Function of the Elderly in Ulm study (ActiFE-Ulm)**

	Database: DEGS1		Database: ActiFE Ulm		
		*Retrospective assessment (12 months)*			*Prospective assessment (12 months)*
Age (years)	Number of participants	Incidence rate of recurrent falls ^†^	Number of participants	Incidence rate of recurrent falls ^†^	Incidence rate of recurrent falls ^†^
**Women**					
65- < 70	400	10.4 (7.2-14.8)	162	8.0 (4.3-13.3)	12.7 (7.9-18.9)
70- < 75	414	10.8 (7.6-15.0)	183	11.5 (7.2-17.0)	8.8 (5.1-14.0)
75- < 80	199	9.7 (5.9-15.4)	110	10.9 (5.8-18.3)	14.1 (8.1-22.2)
80- < 85	-	-	86	23.3 (14.8-33.6)	17.5 (9.9-27.6)
85- < 90	-	-	59	8.5 (2.8-18.7)	20.1 (10.1-33.9)
Total*					
65- < 80	1013	10.4 (8.3-12.9)	455	10.2 (8.5-11.8)	11.6 (9.9-13.2)
65- < 90	-	-	600	11.9 (10.8-13.1)	13.7 (12.5-14.9)
**Men**					
65- < 70	395	5.6 (2.8-10.9)	170	7.1 (3.7-12.0)	8.4 (4.7-13.7)
70- < 75	398	6.1 (3.8-9.7)	240	8.8 (5.5-13.1)	10.5 (6.9-15.3)
75- < 80	180	6.6 (3.5-12.3)	111	9.9 (5.1-17.0)	8.5 (4.0-15.5)
80- < 85	-	-	203	12.3 (8.1-17.6)	13.9 (9.4-19.6)
85- < 90	-	-	64	25.0 (15.0-37.4)	24.2 (13.9-37.2)
Total*					
65- < 80	973	6.1 (4.3-8.5)	521	8.4 (7.1-9.8)	9.3 (7.8-10.7)
65- < 90	-	-	788	10.1 (9.2-11.1)	10.9 (9.9-12.0)

*Incidences weighted according to the age distribution of the German standard population (2010).

^†^%/person-year; 95% confidence interval.

^†^recurrent fallers/100-person-years exposure; 95% confidence interval.

In each study dataset, and for both retrospective and prospective assessment, overall ‘any fall’ incidence rates were consistently higher amongst women than men and the 95% confidence intervals did not overlap. However, for ‘recurrent falls’ the rates did not differ between men and women, nor between study or assessment method, but most confidence intervals overlapped.

### Fall assessment method

There was little or no difference (±2%) in the overall incidence rates obtained by prospective assessment of falls over a 12 month period and retrospective assessment by report of falls over the previous 12 months (Tables 1 and 2). Confidence intervals overlapped between assessments in all overall rates and all age strata for both any fall and recurrent falls. However, it must be noted that for specific age strata the numbers of participants in any one cell are relatively small and consequently the confidence intervals were relatively wide.

## Discussion

We used two different population-based datasets to assess fall incidence in German people aged 65 years and older and living in the community. Fall incidence differed between the two studies. For ‘any fall’, the rate was consistently higher in women than men, and increased with age in men but not consistently so in women. Rates of recurrent falls were similar across the two studies and confidence intervals overlap. There was no difference between retrospectively and prospectively assessed fall incidence rates.

The descriptive data from our two datasets broadly correspond to fall incidence reported in the literature. The LASA study, for example, presented very similar age- and sex-specific fall incidence rates to those observed in the ActiFE-Ulm study [1]. In a survey from the United States which analysed a nationally representative sample of Medicare beneficiaries similar fall rates were observed to DEGS1 [24]. Recently published data from a German telephone survey presented lower fall incidence (17.9% in people aged 65 years and more) [17].

One explanation for the differences in fall incidence estimates for the same target population may be a different selection of people with different fall risks. Since the studies reported here, and Schumacher et al’s [17] telephone survey were designed to be population-based, study design and the way the study population were approached, seem to influence results considerably. The DEGS1 study asked participants to visit a study centre and may therefore represent fall incidence of older people who are still mobile enough to participate; that is a more able and higher functioning section of the population. In the ActiFE-Ulm study assessments were performed at home, which made it easier to participate for persons with mobility restrictions. Even though both studies made considerable efforts to increase participation, the actual participation rates achieved in both studies may be too low to guarantee representativeness in higher ages. For example, persons from DEGS1 aged 65–79 years had a slightly less frequent rate of officially recognized severe disability than persons from the population (20.0% vs. 22.9%) [20]. And people with disabilities in activities of daily living and with care need as assessed by German long-term care insurance criteria, but living at home were underrepresented in the ActiFE-Ulm study (e.g. 0.7% of participants aged 70- < 80 years compared to the 5.1% one would expect based on age specific German population data). Their risk of a femoral fracture is far higher than that of community-living people without assessed care needs, but very similar to that of residents of nursing homes [25]. Therefore, the fall risk of older disabled people living in the community may resemble the very high risks observed in nursing home residents [26, 27] rather than that of community-living people without disabilities. This means that the ‘true’ fall incidence of community-living people could be higher than that presented here. Even though sample sizes of both studies were each clearly larger than 1,000 participants, the statistical power was still too low to compare the estimates of the different strata with each other.

One might expect that the home interview method used in the ActiFE-Ulm study could yield different incidence rates to those obtained using assessment simply by questionnaire, as in the DEGS1. But it is not clear which method would necessarily yield higher rates and which would be closer to the true rate. So the observed differences for ‘any fall’ could be an artefact of method. However, this explanation does not hold true for the assessment of ‘recurrent falls’, where the rates cannot be said to differ between assessment methods.

Collecting falls data using prospective methods is time consuming and costly. Face to face visits by research staff are required to ensure the older person understands how to record falls on a diary or calendar. Whilst returning calendars by post is a relatively modest expense, during the prospective follow up period staff is needed to follow up participants who fail to return calendars, and to contact those who report falls to obtain further data about the fall. The retrospective method can be done during a single interview, or even, as in the DEGS study, by questionnaire. Our results would suggest that one obtains as accurate an estimate of the incidence of falls using a single visit interview as one does using the more costly 12 month prospective approach, although neither can be considered a “gold standard”, and further methods for collection of falls data need to be developed.

There is a risk when retrospectively assessing falls that the data will be inaccurate because of recall biases. One review determined the effect of the recall interval on fall reporting [28]. As expected, longer recall periods resulted in poorer fall reporting particularly in persons with poorer cognitive function. In demented people, recall periods should be even as short as one week and additional information by care-givers is required [29]. Reports of falling in the previous 12 months by people who did not actually fall are rare [28]. In the ActiFE-Ulm study, however, retrospectively assessed data of any fall were somewhat higher in the oldest age-groups. This may be due to impaired time awareness amongst older people, who may mistakenly report fall events from previous years to the last 12 months. Another explanation could be that older people are more willingly to report falls from the past than those from the present since they may be afraid that report of current falls could result in unpleasant consequences like hospitalisation, institutionalisation and loss of independence.

One limitation of our analysis could be that study participants’ retrospectively and prospectively assessed falls did not cover the same time period. But the problem with having a retrospective assessment which covers the same time period as prospective assessment is that participants would be primed to recall falls over that period. Therefore, using two subsequent time periods may actually be a more reasonable way to compare retrospective and prospective assessed fall incidence, although it assumes that the incidence rate remains steady over the two time periods.

A further limitation is that both prospectively and retrospectively assessed falls relied on self-report, which was not verified by such methods as crosschecking with medical records for injurious falls. Previous studies have validated reported falls by checking for other data to confirm and suggest that the method is reliable. To date there is a paucity of data on actual falls, and the literature is dependent on self-report methods and there is no gold standard to provide criterion validity. However, this is changing fast and the use of body worn or body fixed fall sensors with high sensitivity and specificity in community based epidemiological studies is becoming a reality [30]. Other technological solutions include use of video recording and other environment sensors to detect and record falls [31]. Clearly such instrumented falls offer promise for future research, but even they depend on report methods being used at least during the methodological validation stage and more research is required to determine reliability and validity of instrumented falls alongside self-report.

## Conclusion

In conclusion, fall incidence among community dwelling people aged 65 years and older was assessed in two population-based German studies, and this gave us the opportunity to also compare different fall assessment methods. Overall fall incidence of ‘any fall’ assessed retrospectively, but not recurrent fall rates, differed between the two studies, consistent with both the very different possibilities that true rates differed between the two populations and that they were artificial differences because of methodological differences between studies. In the ActiFE-Ulm study, retrospective and prospective methods to assess fall incidence over different assessment periods provided strikingly similar results.

Population-based studies are likely to underestimate true fall incidence among community residents as they have difficulty capturing high risk groups such as physically and cognitively impaired persons, and those depending on home care. Nevertheless, incidence rates of ‘recurrent falls’ observed for the German population residing in the community are considerable and deserve further investigation with respect to determinants and health consequences.

## References

[1] Tromp AM, Pluijm SM, Smit JH, Deeg DJ, Bouter LM, Lips P (2001). Fall-risk screening test: a prospective study on predictors for falls in community-dwelling elderly. J Clin Epidemiol.

[2] Roy DK, Pye SR, Lunt M, O'Neill TW, Todd C, Raspe H, Reeve J, Silman AJ, Weber K, Dequeker J, Jajic I, Stepan J, Delmas PD, Marchand F, Reisinger W, Banzer D, Felsenberg D, Janott J, Kragl G, Scheidt-Nave C, Felsch B, Raspe H, Matthis C, Lyritis G, Poor G, Gennari C, Pols HA, Falch JA, Miazgowski T, Hoszowski K (2002). Falls explain between-center differences in the incidence of limb fracture across Europe. Bone.

[3] Vellas BJ, Wayne SJ, Garry PJ, Baumgartner RN (1998). A two-year longitudinal study of falls in 482 community-dwelling elderly adults. J Gerontol A Biol Sci Med Sci.

[4] O’Loughlin JL, Robitaille Y, Boivin JF, Suissa S (1993). Incidence of and risk factors for falls and injurious falls among the community-dwelling elderly. Am J Epidemiol.

[5] Lord SR, Ward JA, Williams P, Anstey KJ (1993). An epidemiological study of falls in older community-dwelling women: the Randwick falls and fractures study. Aust J Public Health.

[6] Johnson SJ (2006). Frequency and nature of falls among older women in India. Asia Pac J Public Health.

[7] Gillespie LD, Robertson MC, Gillespie WJ, Sherrington C, Gates S, Clemson LM, Lamb SE (2012). Interventions for preventing falls in older people living in the community. Cochrane Database Syst Rev.

[8] Peel NM (2011). Epidemiology of falls in older age. Can J Aging Rev Can Vieil.

[9] Morrison A, Fan T, Sen SS, Weisenfluh L (2013). Epidemiology of falls and osteoporotic fractures: a systematic review. Clinicoecon Outcomes Res.

[10] Tinetti ME, Speechley M, Ginter SF (1988). Risk factors for falls among elderly persons living in the community. N Engl J Med.

[11] Campbell AJ, Reinken J, Allan BC, Martinez GS (1981). Falls in old age: a study of frequency and related clinical factors. Age Ageing.

[12] Nevitt MC, Cummings SR, Kidd S, Black D (1989). Risk factors for recurrent nonsyncopal falls. A prospective study. JAMA.

[13] Chu LW, Chi I, Chiu AYY (2005). Incidence and predictors of falls in the chinese elderly. Ann Acad Med Singapore.

[14] Kojima S, Furuna T, Ikeda N, Nakamura M, Sawada Y (2008). Falls among community-dwelling elderly people of Hokkaido, Japan. Geriatr Gerontol Int.

[15] Tolonen H, Helakorpi S, Talala K, Helasoja V, Martelin T, Prättälä R (2006). 25-year trends and socio-demographic differences in response rates: Finnish adult health behaviour survey. Eur J Epidemiol.

[16] Hauer K, Lamb SE, Jorstad EC, Todd C, Becker C (2006). Systematic review of definitions and methods of measuring falls in randomised controlled fall prevention trials. Age Ageing.

[17] Schumacher J, Pientka L, Trampisch U, Moschny A, Hinrichs T, Thiem U (2014). The prevalence of falls in adults aged 40 years or older in an urban, German population : Results from a telephone survey. Z Gerontol Geriatr.

[18] Scheidt-Nave C, Kamtsiuris P, Gößwald A, Hölling H, Lange M, Busch MA, Dahm S, Dölle R, Ellert U, Fuchs J, Hapke U, Heidemann C, Knopf H, Laussmann D, Mensink GB, Neuhauser H, Richter A, Sass AC, Rosario AS, Stolzenberg H, Thamm M, Kurth BM (2012). German health interview and examination survey for adults (DEGS) - design, objectives and implementation of the first data collection wave. BMC Public Health.

[19] Denkinger MD, Franke S, Rapp K, Weinmayr G, Duran-Tauleria E, Nikolaus T, Peter R, ActiFE Ulm Study Group (2010). Accelerometer-based physical activity in a large observational cohort–study protocol and design of the activity and function of the elderly in Ulm (ActiFE Ulm) study. BMC Geriatr.

[20] Kamtsiuris P, Lange M, Hoffmann R, Schaffrath Rosario A, Dahm S, Kuhnert R, Kurth BM (2013). The first wave of the German Health Interview and Examination Survey for Adults (DEGS1): sample design, response, weighting and representativeness. Bundesgesundheitsblatt Gesundheitsforschung Gesundheitsschutz.

[21] Bellach BM, Knopf H, Thefeld W (1998). The German Health Survey. 1997/98. Gesundheitswesen.

[22] Lamb SE, Jørstad-Stein EC, Hauer K, Becker C, Prevention of Falls Network Europe and Outcomes Consensus Group (2005). Development of a common outcome data set for fall injury prevention trials: the Prevention of Falls Network Europe consensus. J Am Geriatr Soc.

[23] *Statistisches Bundesamt Deutschland - GENESIS-Online*. 2014. Available at: https://www-genesis.destatis.de/genesis/online

[24] Shumway-Cook A, Ciol MA, Hoffman J, Dudgeon BJ, Yorkston K, Chan L (2009). Falls in the Medicare population: incidence, associated factors, and impact on health care. Phys Ther.

[25] Rapp K, Becker C, Cameron ID, Klenk J, Kleiner A, Bleibler F, König HH, Büchele G (2012). Femoral fracture rates in people with and without disability. Age Ageing.

[26] Rubenstein LZ, Josephson KR, Robbins AS (1994). Falls in the nursing home. Ann Intern Med.

[27] Rapp K, Becker C, Cameron ID, König H-H, Büchele G (2012). Epidemiology of falls in residential aged care: analysis of more than 70,000 falls from residents of bavarian nursing homes. J Am Med Dir Assoc.

[28] Ganz DA, Higashi T, Rubenstein LZ (2005). Monitoring falls in cohort studies of community-dwelling older people: effect of the recall interval. J Am Geriatr Soc.

[29] Zieschang T, Schwenk M, Becker C, Oster P, Hauer K (2012). Feasibility and accuracy of fall reports in persons with dementia: a prospective observational study. Int Psychogeriatr.

[30] Schwickert L, Becker C, Lindemann U, Maréchal C, Bourke A, Chiari L, Helbostad JL, Zijlstra W, Aminian K, Todd C, Bandinelli S, Klenk J, FARSEEING Consortium and the FARSEEING Meta Database Consensus Group (2013). Fall detection with body-worn sensors: a systematic review. Z Gerontol Geriatr.

[31] Robinovitch SN, Feldman F, Yang Y, Schonnop R, Leung PM, Sarraf T, Sims-Gould J, Loughin M (2013). Video capture of the circumstances of falls in elderly people residing in long-term care: an observational study. Lancet.

[CR32] The pre-publication history for this paper can be accessed here: http://www.biomedcentral.com/1471-2318/14/105/prepub

